# Plea for routine endoscopic tracheostomy tube adjustment

**DOI:** 10.3389/fresc.2025.1598300

**Published:** 2025-07-24

**Authors:** Bettina Otto, Regina Lindemann, Holger Kirsch, Matthias Schmid, Hartmut Vatter, Christiane Braun

**Affiliations:** ^1^ Early Rehabilitation Department, Neurological Rehabilitation Center Godeshöhe, Bonn, Germany; ^2^ Institute for Medical Biometry, Informatics and Epidemiology (IMBIE), University Hospital Bonn, Bonn, Germany; ^3^ Department of Neurosurgery, University Hospital Bonn, Bonn, Germany

**Keywords:** tracheostomy tube adjustment, tracheal cannula management, tracheal lesion, decannulation, tracheoscopy

## Abstract

**Introduction:**

Tracheostomy is one of the standard procedures in intensive care medicine. In the context of tracheostomy tube-, dysphagia- and decannulation management the selection of the appropriate tracheostomy tube model (angle, diameter, length) is crucial for the proper placement in the trachea. In spite of recent guidelines mentioning endoscopic control of the tube placement as a useful measure, data regarding the proper placement are rare in the present literature. Therefore, the aim of the present study was to investigate the accuracy of tracheostomy tube placement in patients admitted to our early neurological rehabilitation center.

**Methods:**

We performed a retrospective single-center analysis of all patients with tracheostomy tube admitted to our early neurological rehabilitation center between 12/2022 and 01/2024. We analyzed the frequency, type and extent of injuries caused by a suboptimal placement of the tracheostomy tubes. The location of the tubes was routinely controlled endoscopically upon admission. In total 327 tracheoscopies were carried out. Clinical characteristics were collected in all patients and the endoscopic results were divided into malpositioned tracheostomy tubes (non-central tube position, often causing mucosal lesions, ulcer, bleeding) vs. well-positioned (central or almost central) tubes. The association between the quality of the tracheostomy tube placement and the characteristics age, gender, main diagnosis, tracheostomy procedure, time until initial endoscopic control of tracheostomy tube fitting after admission and after tracheostomy were analyzed using a logistic regression model.

**Results:**

A total of 214 examinations (65%) revealed a malpositioned tracheostomy tube. In 19% of the carried out tracheoscopies (327), manifest injuries were already detectable (mucosal lesion, ulcer, bleeding). 113 examinations (35%) showed an acceptable tube placement. We found no association between the quality of the tracheostomy tube position and gender, age, main diagnosis, time until initial endoscopic control of tube fitting or type of tracheostomy.

**Discussion:**

Since we found a high percentage of suboptimal tracheostomy tube positions (65%), an increased risk of complications can be assumed. With a view to the most relevant late complication of tracheal stenosis, there is agreement that the fundamental lesion begins with mucosal ulceration, which we found in 19% of the investigations. Therefore, the present data strongly suggest that a routine endoscopic control of tracheostomy tube placement should be firmly implemented into the routine tracheostomy tube management. Our data further suggest that the supply with tracheostomy tubes needs to be optimized.

## Introduction

With over 44.000 procedures in Germany in 2022 tracheostomy is one of the standard procedures in the field of intensive care and respiratory medicine in cases of prolonged respiratory weaning and severe dysphagia (1).

As beneficial as tracheostomy proves to be in the acute phase of the disease, are the numerous and wide-ranging aspects that arise on the way to decannulation. From a multidimensional perspective, successful decannulation is of extraordinary importance. In addition to individual patient-related aspects such as quality of life (2–4) and the identification of missing decannulation as negative outcome parameter (5, 6), economic aspects are certainly also of concern.

In the context of tracheostomy tube-, dysphagia- and decannulation management the selection of the appropriate tracheostomy tube model (angle, diameter, length)—according to clinical experience—is crucial for the proper placement in the trachea (7).

Suboptimally placed tracheostomy tubes can affect ventilation and breathing as well as secretion management, cause relevant injuries to the trachea and complicate the dysphagia management.

As early as the 1960s, post-mortem examinations demonstrated the effects of the tracheostomy itself as well as the placed tracheostomy tubes and described any injury patterns caused by this. Infrastomal lesions, Cuff pressure-associated lesions or injuries caused by the tracheostomy tube resting against the tracheal anterior or posterior wall resulting in wounds, fistulas, malacia or secondary stenosing processes were described (8). A prospective study from this time confirmed these connections and found functional relevant tracheal stenosis in nearly 20% of the examined tracheostomized patients (9).

Regardless of possible secondary damage caused by the tracheostomy itself or the quality of the tube placement, the correct position of the tracheostomy tube can only be verified by endoscopic control.

Accordingly, the recommendation made in both the current guideline “Neurogenic Dysphagia” and in corresponding overview articles is to regularly check the tracheostomy tube placement in tracheostomized patients (7, 10).

Although the endoscopic control of the tracheostomy tube position is mentioned ubiquitous, there are currently no uniform standards for the detailed procedure.

Management aspects of tracheostomies such as an optimal tube management are typically driven by individual expertise and local preferences. Real quality indicators are not yet established.

To the best author's knowledge, there is a lack of reliable data, for example on the frequency, type and extent of any injuries caused by a suboptimal tracheostomy tube placement, which would justify the recommendation beyond mere empirical medicine.

The aim of the present data is to provide evidence for the often-made recommendation of regular endoscopic checks during tracheostomy tube management to ensure optimally positioned tracheostomy tubes.

## Materials and methods

We conducted an exploratory, retrospective, observational study at a neurorehabilitation center.

According to the statement of the Ethics Committee of The North Rhine State Chamber of Physicians (5-2005) a formal consent was not necessary because of the retrospective study design.

The study was performed following the Declaration of Helsinki. Informed consent was waived due to the retrospective nature of the analysis. Data analysis had no impact on participants or their medical care.

**Figure 1:**
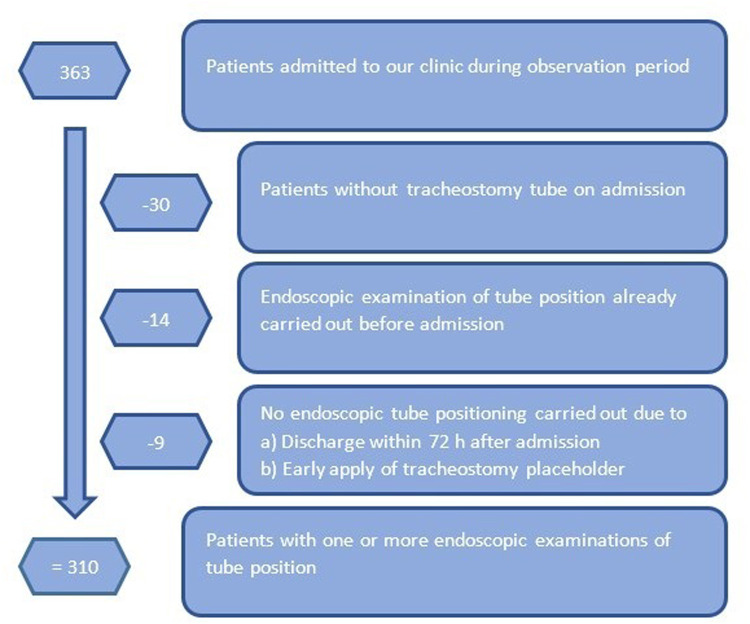
Flowchart illustrating the selection process of included patients.

For this purpose, we screened all 363 patients admitted to our early neurological rehabilitation center during the period from December 2022 to January 2024 for the presence of an inserted tracheostomy tube. We excluded patients admitted without tracheostomy tube, those having received endoscopic tracheostomy tube positioning before admission to our clinic already and patients ready to receive a tracheostomy placeholder immediately. Discharge during the first 72 h after admission was also an exclusion criterion (Figure 1).

All remaining tracheostomized patients had regular endoscopic checks of the tracheostomy tube position during tracheostomy tube- and dysphagia management. Therefore, a fiberoptic endoscope was passed via the tube to visualize the cannula's distal tip and the tracheal lumen around it. When the clinical condition allowed, the cannula was completely removed to assess the entire tracheostomy and the tracheal lumen. The endoscopic examination was carried out by specialized rehabilitation physicians in collaboration with speech therapists. If necessary (malpositioned tube verified), the tracheostomy tube model was changed, and the newly inserted tube was endoscopically re-checked for optimal fit.

**Figure 2:**
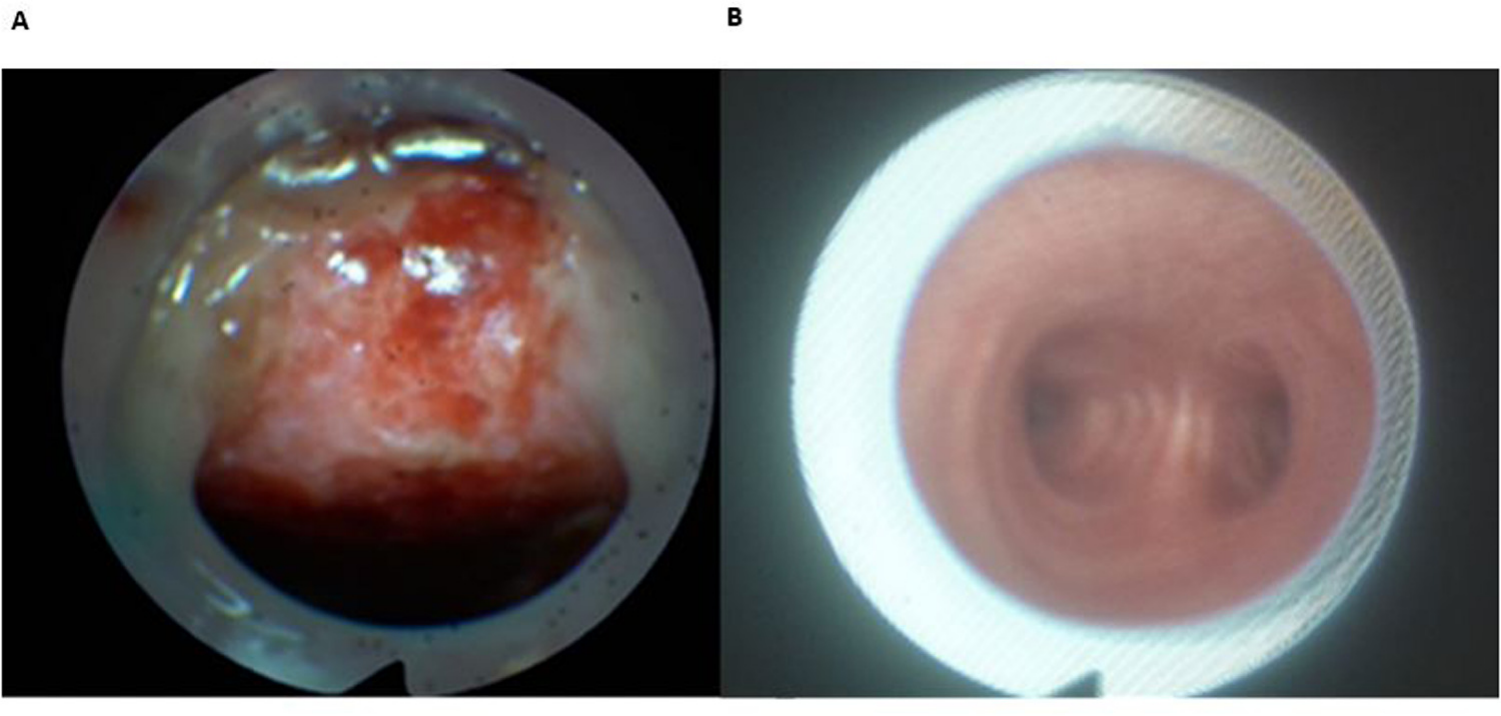
Malpositioned **(A)** vs. well-positioned **(B)** tracheostomy tube. **(A)** Malpositioned tube; visible ulcus of tracheal posterior wall. **(B)** Well-positioned tube; central located tube with view of the bifurcation.

Data were collected from the neurorehabilitation center electronic medical records, chart systems and the Fiberoptic Endoscopic Evaluation of Swallowing (FEES)-database. We describe the initial tracheostomy tube placement. The results were categorised in two possible categories (Figure 2):

1. Malpositioned (non-central) tracheostomy tube: Cannula lies in the tracheal wall, in part with already recognizable injury [visible bleeding or tissue damage (ulcer/necrosis)]. Tracheostomy tube position needs correction.
2. Well-positioned (central or almost central) tracheostomy tube: Tracheostomy tube model is retained or is changed only to improve physiological airflow guidance by using a smaller cannula model (planned downsizing).

The treatment decisions made based on the examinations were documented using an evaluation form.

### Statistical analysis

Continuous variables were analyzed using means with standard deviations (SDs) and medians with minimum & maximum values. Categorical variables were analyzed using counts with percentages. Bivariate associations between tube position and (i) age, (ii) gender, (iii) main diagnosis, (iv) tracheostomy procedure, (v) time to initial endoscopic investigation after admission and (vi) time up to initial endoscopic investigation after tracheostomy were analyzed by Mann–Whitney *U* tests, chi-squared tests and Fisher's exact tests, as appropriate. Logistic regression was used for multivariable analysis (dependent variable: tube position). Two-sided *p*-values < 0.05 were considered significant. Missing values (<1%, see Table 1) were excluded from statistical analysis. All measurements of tube position were considered as independent, regardless of whether obtained from the same patient or not. This assumption was justified by the fact that in these cases anatomical conditions changed due to tracheostomy conversion, a surgical intervention due to tracheal stenosis or an unsuccessful decannulation trial in the meantime and therefore justified another tracheostomy tube adjustment.

**Table 1:** Patient characteristics of the 310 patients included in the data analysis.

Patient characteristics	Malpositioned tube (Category 1) *N* = 206	Well-positioned tube (Category 2) *N* = 104	Patients in total *N* = 310
Age (years)			
Mean (SD)	64.4 (12.7)	63.0 (12.4)	64.0 (12.6)
Median (Min; Max)	66.0 (22.0; 88.0)	63.0 (16.0; 88.0)	65.0 (16.0; 88.0)
Missing	1 (0.5%)	0 (0%)	1 (0.3%)
Gender			
Male	134 (65.0%)	63 (60.6%)	197 (63.5%)
Female	72 (35.0%)	41 (39.4%)	113 (36.5%)
Main diagnosis			
Cerebral hemorrhage	63 (30.6%)	24 (23.1%)	87 (28.1%)
Ischaemic stroke	48 (23.3%)	33 (31.7%)	81 (26.1%)
Traumatic brain injury	24 (11.7%)	10 (9.6%)	34 (11.0%)
Inflammatory disease	10 (4.9%)	2 (1.9%)	12 (3.9%)
Guillain-barré-syndrome	4 (1.9%)	3 (2.9%)	7 (2.3%)
Hypoxic encephalopathy	22 (10.7%)	11 (10.6%)	33 (10.6%)
ICUaW/PICS	21 (10.2%)	14 (13.5%)	35 (11.3%)
Muscle diseases	7 (3.4%)	0 (0%)	7 (2.3%)
Neoplasms	2 (1.0%)	3 (2.9%)	5 (1.6%)
Other	5 (2.4%)	4 (3.8%)	9 (2.9%)
Type of tracheostomy			
Surgical tracheostomy	52 (25.2%)	26 (25.0%)	78 (25.2%)
Percutaneous dilated tracheostomy	154 (74.8%)	78 (75.0%)	232 (74.8%)
Tube adjustment after tracheostomy (days)			
Mean (SD)	25.7 (16.6)	27.3 (16.6)	26.2 (16.6)
Median (Min; Max)	21.0 (3.0; 131.0)	23.5 (7.0; 89.0)	21.0 (3.0; 131.0)
Tube adjustment after admission (days)			
Mean (SD)	4.6 (4.2)	4.0 (3.9)	4.4 (4.1)
Median (Min; Max)	4.0 (0.0; 35.0)	3.0 (0.0; 22.0)	3.00 (0.0; 35.0)

Category 1: Tracheostomy tube malpositioned (non-central, needing correction). Category 2: Tracheostomy tube well-positioned (central, no correction needed).

## Results

According to the criteria above 310 (92% of all) tracheostomized patients were included in the analysis. Only 30 patients had no tube at the time of initial admission. All patients included received routine endoscopic checks of the tracheostomy tube position during tracheostomy tube- and dysphagia management. If necessary, the tracheostomy tube model was changed (Figure 1).

Fifteen patients had double examinations and one was examined three times, since the originally adapted tracheostomy tube was changed or the tracheostomy revised as part of the transfer to another hospital. Therefore, we carried out 327 endoscopic examinations (Table 2) on 310 patients (Table 1) during the investigation period. Tracheostomy tube position was categorized in malpositioned (Category 1) vs. well-positioned (Category 2) placement.

**Table 2:** Patient characteristics of the 327 tracheoscopies included in the data analysis.

Patient characteristics	Malpositioned tube (Category 1) *N* = 214	Well-positioned tube (Category 2) *N* = 113	Tracheoscopies in total *N* = 327
Age (years)			
Mean (SD)	64.4 (12.7)	63.5 (12.2)	64.1 (12.5)
Median (Min; Max)	65.0 (22.0; 88.0)	65.0 (16.0; 88.0)	65.0 (16.0; 88.0)
Missing	1 (0.5%)	0 (0%)	1 (0.3%)
Gender			
Male	136 (63.6%)	68 (60.2%)	204 (62.4%)
Female	78 (36.4%)	45 (39.8%)	123 (37.6%)
Main diagnosis			
Cerebral hemorrhage	64 (29.9%)	26 (23.0%)	90 (27.5%)
Ischaemic stroke	49 (22.9%)	35 (31.0%)	84 (25.7%)
Traumatic brain injury	25 (11.7%)	12 (10.6%)	37 (11.3%)
Inflammatory disease	10 (4.7%)	2 (1.8%)	12 (3.7%)
Guillain-barré-Syndrome	5 (2.3%)	3 (2.7%)	8 (2.4%)
Hypoxic encephalopathy	22 (10.3%)	13 (11.5%)	35 (10.7%)
ICUaW/PICS	24 (11.2%)	14 (12.4%)	38 (11.6%)
Muscle disease	8 (3.7%)	0 (0%)	8 (2.4%)
Neoplasms	2 (0.9%)	3 (2.7%)	5 (1.5%)
Other	5 (2.3%)	5 (4.4%)	10 (3.1%)
Type of tracheostomy			
Surgical tracheostomy	57 (26.6%)	33 (29.2%)	90 (27.5%)
Percutaneous dilational tracheostomy	157 (73.4%)	80 (70.8%)	237 (72.5%)
Tube adjustment after tracheostomy (days)			
Mean (SD)	25.7 (17.3)	26.7 (17.9)	26.0 (17.5)
Median (Min; Max)	20.5 (3.0, 131.0)	22.0 (2.0; 91.0)	21.0 (2.0; 131.0)
Missing	2 (0.9%)	0 (0%)	2 (0.6%)
Tube adjustment after admission (days)			
Mean (SD)	4.6 (4.2)	3.9 (3.9)	4.4 (4.1)
Median (Min; Max)	4.0 (0.0; 35.0)	3.0 (0.0; 22.0)	3.0 (0.0; 35.0)
Missing	6 (2.8%)	7 (6.2%)	13 (4.0%)

Category 1: Tracheostomy tube malpositioned (non-central, needing correction). Category 2: Tracheostomy tube well-positioned (central, no correction needed).

Baseline characteristics of the included 310 patients are given in Table 1.

At initial endoscopic examination the average age of the included patients was 64.0 years (SD = 12.6 years). Regarding the gender distribution, there was a clear predominance of male patients, in a ratio of 63.5% men to 36.5% women. As expected, the percutaneous tracheostomy procedure clearly predominated with 75% vs. 25% surgical tracheostomies (Table 1).

Regarding the distribution of diagnosis, cerebral vascular diseases and TBI accounted for almost two thirds of all cases examined (202 patients, 65%). Following cerebral hemorrhage and cerebral ischaemic stroke, traumatic brain injury (TBI) was the fourth most common diagnosis, numerically almost equal to the number of patients suffering from Intensive Care Unit-Acquired Weakness (ICU-aW)/post-Intensive-Care-Syndrome (PICS) and hypoxic-ischaemic encephalopathy (about 11% each, Table 1).

The initial endoscopic investigation with tracheostomy tube adjustment if necessary took place on average 4.4 days after admission in our rehabilitation center (Median 3 days, Minimum 0 days, Maximum 35 days) and 26.2 days after tracheostomy (Median 21 days, Minimum 3 days, Maximum 131 days).

In total 214 examinations met the criteria for Category 1 with malpositioned tracheostomy tube, corresponding to 65% of the examinations. Visible relevant lesions of the tracheal wall (ulcus, bleeding, lesion of the tracheal mucosa) were detectable in 19% of all tracheoscopies (61/327) carried out. All malpositioned tracheostomy tubes were replaced with another model, the correct position of which was checked by endoscopy.

The tracheostomy procedure itself [percutaneous dilational tracheostomy with malpositioned tracheostomy tube in 157/237 investigations (66%) vs. surgical tracheostomy with malpositioned tracheostomy tube in 57/90 investigations (63%)] did not show any association with central or non-central tube location (*p* = 0.716).

We found no association between the need for correction of the tracheostomy tube position (Category 1) and gender, just as little as between the need for correction of the cannula position and the terms of age, main diagnosis, time to initial endoscopic investigation after admission, and time up to initial endoscopic investigation after tracheostomy (see Table 2). When the characteristics were analyzed jointly in a multivariable logistic regression model, no connections were found either.

When the initial endoscopic assessment of the cannula location took place at a relatively early point in time after tracheostomy, defined as ≤16 days after tracheostomy (regardless of the type of procedure), a non-central located cannula in need of correction was found in 64% of cases (74 out of 115 investigations). If the examination was carried out >16 days after tracheostomy it was assigned to Category 1 (malpositioned cannula) in 66% of cases (138 out of 210 investigations, *p* = 0.90).

## Discussion

Since tracheostomy is a standard procedure in intensive care medicine and patients with a neurological main diagnosis have higher tracheostomy rates than those with a non-neurological main diagnosis (11), the high proportion of tracheostomized patients (92%) in the neurological early rehabilitation clientele examined here is hardly surprising.

Our data revealed a malposition of the inserted tracheostomy tubes in two thirds of the carried out endoscopic examinations (214/327 = 65%). Sixty-one of 327 investigations (19%) confirmed visible injuries of the trachea. This partly leads to problems in airway management like difficult ventilation or difficulties with physiological breathing via mouth and nose having the cuff deflated and the cannula temporarily closed with a cap that may result in insufficient dysphagia therapy. Besides, it can also cause patterns of injury that, according to data and clinical experience, can at least promote the occurrence of tracheal stenosis (9, 8, 12).

Undeniably, at the beginning of acute early rehabilitation, an essential multidisciplinary treatment focus is the diagnosis and treatment of the swallowing disorder in the course of tracheostomy tube management. Main goal is successful decannulation (13), which largely determines the probability and quality of survival (5). In their longitudinal data on this subject, Pohl and colleagues were able to show that probability of survival decisive depends on the functional status at the time of discharge from early neurological rehabilitation. Patients discharged with a tracheostomy tube have a significantly reduced probability of survival (6). These data strongly support, that respiration and swallowing ability is a central topic that determinates the outcome.

Since the prolonged supply with a tracheostomy tube means a significant loss of quality of life (2–4)*,* successful decannulation is of utmost importance and the next therapy step after weaning from the respirator has been completed successfully (14). In this context, our data emphasize the importance of choosing the appropriate tracheostomy tube model within the tube-, dysphagia- und decannulation-management.

As the negative effects of suboptimally placed tracheostomy tubes are well known and a malpositioned tracheostomy tube can only be detected by endoscopic investigation, this procedure is recommended in the current guideline “Neurogenic Dysphagia” as well as in review articles addressing tube weaning (7, 10).

In the literature, the connection between tracheal lesions caused by tube placement and resulting complications is postulated and often discussed (9, 15, 8).

Already 1969 Cooper and Grillo examined the negative effects of endotracheal tubes and tracheostomy tubes on the tracheal morphology post mortem. They identified different kind of lesions due to suboptimal cannula placement and/or high Cuff-pressure followed by some inflammatory reaction and development of granulation tissue or scar-like reaction causing secondary tracheal stenosis (8, 12). Another prospective study showed of comparable results with 17.5% of patients presenting with tracheal stenosis after mechanical ventilation and tracheostomy (9). In some patients, predisposing tracheal ulcerations could be detected endoscopically prior to the development of the stenosis.

Matching these long-known data Ledl and colleagues recommend endoscopic control of tracheostomy tube position due to postulated preventive effects regarding laryngo-tracheal stenosis or development of tracheal granulation tissue, whose origin is most likely due to irritation caused by tracheostomy tubes followed by chronic inflammation of the tissue (10). These judgement corresponds to the older publications mentioned above (8, 9).

The occurrence of stenosing granulation tissue and stricture-related tracheal stenosis of various locations after tracheostomy also largely corresponds to the authoŕs own experiences.

Despite optimization of both the tracheostomy tube material and major advances in tracheostomy tube management overall, our data show that a malpositioned tracheostomy tube often occurs. Based on the available data and results, the working group believes that endoscopically controlled tube placement can improve the quality of tracheal tube management, potentially accelerating decannulation and helping prevent complications, even though our retrospective data cannot conclusively prove these outcome benefits. Whether routine endoscopic control of tracheostomy tube position has a positive effect on the time to decannulation or helps prevent complications should be subject to future investigation.

The frequency of suboptimal inserted tracheostomy tubes after tracheostomy allows us to conclude that endoscopic checking of the cannula position should be an obligatory part of tracheostomy tube management. The fact that the suboptimal position of the tracheostomy tube—demonstrated in 65% of the analyzed examinations—can promote the formation of granulation tissue with subsequent stenosis seems logical and obvious, but cannot be proven based on the available data. The establishment of a comparison group in the sense of a prospective study design does not appear to be ethically justifiable, so the historical data mentioned support this connection.

Interesting is the fact that in our data set, the duration from tracheostomy to tracheoscopy (≤16 days vs. >16 days after tracheostomy; 64% vs. 66% malpositioned cannula) did not show any relevant association with the quality of the tube fit, nor did the tracheostomy procedure itself (percutaneous tracheostomy vs. surgical tracheostomy; 66% vs. 63% with malpositioned tube). This can probably be explained by the fact that no tracheostomy tube change took place before admission to our rehabilitation center, the change was not carried out under endoscopic control, or that many hospitals do not have a range of different tube models or appropriately trained personnel.

Possibly helpful would be the routine assessment of the tube placement as part of the standardized tracheostomy-protocol, especially with reference to the need for a timely optimization of the tube fit. In view of possible risks associated with early tracheostomy tube changes, especially in case of percutaneous tracheostomies, we recommend a routine position check as part of the first tracheostomy tube change or upon admission to the neurological rehabilitation center.

In view of existing gaps in knowledge and to confirm the results presented, it is worthwhile to conduct a prospective multicenter examination regarding tube placement and predictors of successful decannulation. In Germany, recruiting study centers along the structure of DGNR-certified weaning centers would be a logical approach.

### Limitations

The retrospective and single-center design of this study is clearly a limitation.

Detailed information of bronchoscopy results of the referring clinics reflecting the tracheostomy tube placement are not routinely reported, so it could not be analyzed in this study.

## Conclusions

We found a poorly fitting tracheostomy tube in well over half of the patients examined, with visible consequential damage in 19% of the cases. This can result in difficulties in breathing, ventilation or pain and coughing. In addition, the development of granulation tissue formation or scar changes following tracheal lesions is one of the favoured primary mechanism of tracheal stenosis. Taking these facts into account endoscopic control of the tracheostomy tube placement should be established as a central component of effective tracheostomy tube- and dysphagia management mandatorily. Positive results can be expected both for acute management and with regard to long-term complications.

The time and material expenditure of this measure appears justifiable.

## Data Availability

The original contributions presented in the study are included in the article/Supplementary Material, further inquiries can be directed to the corresponding author.
